# Explainable Machine Learning for Detecting Pancreatic Cancer from Structured Endoscopic Ultrasound Data: A Retrospective Multicenter Observational Study

**DOI:** 10.3390/jcm15156094

**Published:** 2026-08-05

**Authors:** Nunzio Zignani, Marco Balzarini, Gloria Lopiano, Andrea Campagner, Emanuele Dabizzi, Elia Fracas, Laura Millefanti, Sergio Segato, Gianpaolo Cengia, Vincenzo Villanacci, Guido Missale, Maurizio Vecchi, Gian Eugenio Tontini, Dario Moneghini, Federico Cabitza, Flaminia Cavallaro

**Affiliations:** 1 Department of Pathophysiology and Transplantation, University of Milan, 20122 Milan, Italy; 2 Gastroenterology and Digestive Endoscopy Unit, ASST Sette Laghi, 21100 Varese, Italy; 3 Department of Computer Science, Systems and Communication, University of Milano-Bicocca, 20126 Milan, Italy; 4 IRCCS Ospedale Galeazzi Sant’Ambrogio, 20157 Milan, Italy; 5 Gastroenterology and Endoscopy Unit, Fondazione IRCCS Ca’ Granda, Ospedale Maggiore Policlinico, 20122 Milan, Italy; 6 Digestive Endoscopy Unit, ASST Spedali Civili, Street, 25123 Brescia, Italy; 7 Department of Pathology, ASST Spedali Civili, Street, 25123 Brescia, Italy

**Keywords:** pancreatic ductal adenocarcinoma (PDAC), pancreatic cancer, early PDAC detection, pancreatic solid lesions, explainable machine learning, predictive models

## Abstract

**Background**: Machine learning (ML) is increasingly applied in medicine, underscoring the need for transparent and clinically relevant models. In gastrointestinal oncology, most ML studies rely on raw imaging data, which limits clinical adoption due to poor interpretability and the difficulty of collecting high-quality, large-scale video and image datasets in routine practice. Endoscopic ultrasound (EUS) plays a central role in the evaluation of pancreatic cancer; however, structured EUS features remain underused in predictive modeling. **Objective**: To assess the performance and interpretability of ML models for diagnosing pancreatic ductal adenocarcinoma (PDAC) using routinely collected EUS variables. **Methods**: We conducted a retrospective multicenter study using data from two Italian hospitals (n = 641) for model training and internal validation and from a third hospital (n = 120) for external validation, collected from 2015 to 2023. Decision trees, random forests, naïve Bayes and other classifiers were developed and evaluated. Model performance was assessed in terms of discriminative ability, calibration, and selective prediction. Results: All models demonstrated high discriminative performance (AUC ≥ 0.90). Decision trees provided the most favorable balance between interpretability and accuracy (balanced accuracy = 0.87; sensitivity = 0.89). Calibration and selective prediction analyses confirmed the robustness of the models. **Conclusions**: These findings demonstrate the feasibility of implementing interpretable yet high-performing ML models for PDAC diagnosis in real-life endoscopic settings.

## 1. Introduction

In industrialized countries, pancreatic cancer is a disease that currently represents the tenth most prevalent neoplasm but the third leading cause of cancer-related deaths, surpassed only by lung and colorectal malignancies [1,2]. Unlike almost all other types of cancer, the prognosis of this disease has not improved in recent decades; clinical research is struggling to find a cure, due to its chemo- and radioresistance characteristics [3,4], while early diagnosis is still difficult to achieve, due to the indolent course and the rapid growth and spread to surrounding organs [5,6].

Among the various research frontiers in pancreatic cancer management, artificial intelligence (AI), and specifically so machine learning (ML), has been considered particularly promising due to its ability to automatically analyze large volumes of data [7,8]. Currently, numerous clinical studies in the literature utilize AI models to approach a large number of tasks (e.g., the diagnosis or prognosis of a disease or identification of the best therapeutic strategy [9,10]) and in most cases the applied algorithms are neural networks, which are also employed for multimodal analyses, combining diverse data types, such as clinical, biochemical, and instrumental data, into a unified prediction. These are presented in their general form (Artificial Neural Networks, ANN) or in more specific forms as in the case of image analysis (Convolutional Neural Networks, CNN) [11] and represent the most versatile and performing models despite their greater implementation complexity (as these models generally require dedicated professionals) and low interpretability [12]. The consequence of this is the limitation of AI to clinical research settings only or in tertiary centers, sometimes leading to a distrust of physicians towards this technology, which is too complex for non-experts and not applicable in clinical practice [13].

In the literature, pancreatic tumors, particularly ductal adenocarcinoma (PDAC), have been the subject of numerous applications of ML models, especially in the main diagnostic disciplines such as endosonography (EUS) and radiological examinations such as CT [14,15]. The methods and research questions of these studies are varied; as mentioned above, ANNs and CNNs represent the most used model [16,17,18], but SVMs and RFs are also found [19,20], while the objective to be achieved is generally the diagnosis of PDAC, sometimes in comparison with other lesions [16] or with the analysis of the contrast agent intensity curve [21] or with the study of the elastographic pattern [22]. However, a common limitation of such previous studies that has limited the adoption of ML solutions in clinical practice lies in the fact that they usually rely on raw imaging data or radiomics features, which are hardly interpretable by clinicians and, hence, in turn, hinder the interpretability of the ML models [23]. 

The utility of a model that predicts the presence of PDAC among solid pancreatic lesions lies in the role it can have in supporting the cytological or histological diagnosis following needle biopsy, helping to clarify any complex situations and avoiding examination repetition and the consequent loss of critical time required for a potential surgical resection. The accuracy of this method, in fact, is between 78% and 95%, according to the experience and volume of the center [24,25]. 

The primary objective of this work is to identify the best ML model in predicting the presence of PDAC among solid pancreatic lesions using structured data extracted manually from endosonographic images (without the use of computer-aided diagnosis software). No neural networks will be used but predictive algorithms that could also be implemented and interpreted by a physician, both for research and for supporting clinical practice. Our second aim is to evaluate whether simpler, interpretable models can match or exceed the performance of more complex approaches. Additionally, we seek to understand how well these models handle uncertainty and whether their predictions are reliable enough to support real-world diagnostic decisions.

## 2. Materials and Methods

### 2.1. Study Design

This study is a retrospective, multicenter, observational investigation conducted across three tertiary-care centers in Northern Italy: Fondazione IRCCS Ca’ Granda–Ospedale Maggiore Policlinico di Milano, ASST Spedali Civili di Brescia, and ASST dei Sette Laghi di Varese. The study cohort comprises patients who underwent endoscopic ultrasound (EUS) between 2015 and 2023 for the evaluation and staging of a previously detected solid pancreatic lesion. Initial identification of lesions was performed using standard cross-sectional imaging modalities, namely abdomen ultrasound, computed tomography (CT) or magnetic resonance imaging (MRI). The instruments used were Pentax EG-3870UTK and Pentax EG-38J10UT for echoendoscope (Pentax Medical, Tokyo, Japan) and Hitachi Arietta v70 and Hitachi Hi Vision Avius^®^ for sonograph (Fujifilm Healthcare, Tokyo, Japan). All patients underwent needle biopsy (fine needle aspiration—FNA/fine needle biopsy—FNB) performed using a 19-22-25 gauge Expect™ and Acquire™ (Boston Scientific, Marlborough, MA, USA) and EchoTip Pro-Core^®^ (Cook Medical, Bloomington, IN, USA). The endoscopic ultrasound procedure was harmonized at the three participating centers in accordance with the European Society of Gastrointestinal Endoscopy (ESGE) guidelines [26,27]. The EUS-FNA and EUS-FNB procedures involved visualization of the lesion with a linear echoendoscope, followed by transduodenal or transgastric insertion of the fine needle. Once the echoendoscope was optimally positioned, the needle was advanced through the instrument channel and into the target lesion. Adequate tissue acquisition was achieved by performing a “to-and-fro” motion for an average of two to four passes, frequently aided by negative pressure or a stylet. The acquired tissue was subsequently extruded from the needle and submitted for pathological analysis. Diagnoses were established based on cytological and/or histological examination. In cases where histopathological confirmation was not definitive, a clinical and radiological follow-up of at least 12 months was employed to ascertain the diagnosis. To minimize intra-operator variability, all procedures were performed by expert operators (>250 EUS). The EUS data were not collected in the form of raw images or video sequences. Instead, relevant imaging-derived variables were extracted and entered into a structured database using the REDCap data management platform. The dataset was pseudonymized at the point of entry and subsequently anonymized using the ARX anonymization tool to ensure compliance with data protection regulations. Each patient is represented by a single row in the database, while columns comprise demographic information (age and sex), the final binary diagnosis (PDAC versus non-PDAC), and a set of structured ultrasound features.

### 2.2. Data Collection

A total of 761 patients were included in the study, of whom 480 were diagnosed with pancreatic ductal adenocarcinoma (PDAC) and 281 with non-PDAC lesions.

Non-PDAC lesions included neuroendocrine tumors (NET), metastases, focal pancreatitis, rare diagnosis and non-diagnostic findings (generally small solid masses representing the outcomes of previous inflammatory processes).

The distribution of positive and negative cases differed substantially across the three participating hospitals: Brescia contributed 301 positive and 174 negative cases; Policlinico contributed 86 positive and 80 negative cases; and Varese contributed 93 positive and 27 negative cases. A broad descriptive analysis of the sample is available in a previous paper from our group [28]. During the data preparation, no class imbalance reduction strategies were applied. This characteristic of the target variable was managed by the parameters of the classifiers and by taking in more consideration evaluation metrics created for those cases like balanced accuracy and F1 score. Continuous variables included patient age and lesion dimensions (major and minor axes), while categorical features encompassed sex, vascular invasion, lymphadenopathy, ductal invasion, echogenicity, lesion margins, and multiplicity. These features had been encoded as binary variables to facilitate model interpretability and implementation. Arterial invasion and venous invasion indicate the presence (1) or absence (0) of tumor infiltration into adjacent arterial or venous structures, respectively, based on EUS imaging. Vessel compression refers to deformation or narrowing of vascular structures without definitive signs of invasion and is coded as present (1) or absent (0). Lymphadenopathy captures the presence (1) or absence (0) of regional lymph node enlargement, potentially indicative of malignancy. Ductal invasion represents involvement of the main pancreatic duct or the common bile duct, with a value of 1 denoting visible retrodilation. Echogenicity describes the mass’s ultrasound reflectivity; 0 corresponds to a hypoechoic lesion (darker appearance, often suggestive of malignancy), whereas 1 denotes iso- or hyperechoic characteristics. Margins describe the regularity of lesion borders, with 0 indicating irregular, ill-defined edges and 1 corresponding to well-defined, regular contours. Finally, multiple lesions is a binary variable indicating whether more than one pancreatic mass was identified (1) or not (0). Inter-rater agreement was calculated on a sample of 30 patients collected arbitrarily and non-randomly to ensure sufficient representation of less frequent features (e.g., multifocality) in the operators’ assessment of ultrasound images. This parameter was not considered applicable to the B-mode size of the lesion, as the measurement, performed using the “virtual ruler” was present in the reviewed ultrasound images. Data collection also included internal vascularization, stiffness (measured by elastography), and the lesion’s echopattern, all of which were subsequently excluded from model training. Internal vascularization and elastography were excluded because they were available for fewer than half of the patients, whereas echopattern was excluded due to very low inter-rater agreement (calculated for each variable using Krippendorff’s Alpha).

**Figure 1:**
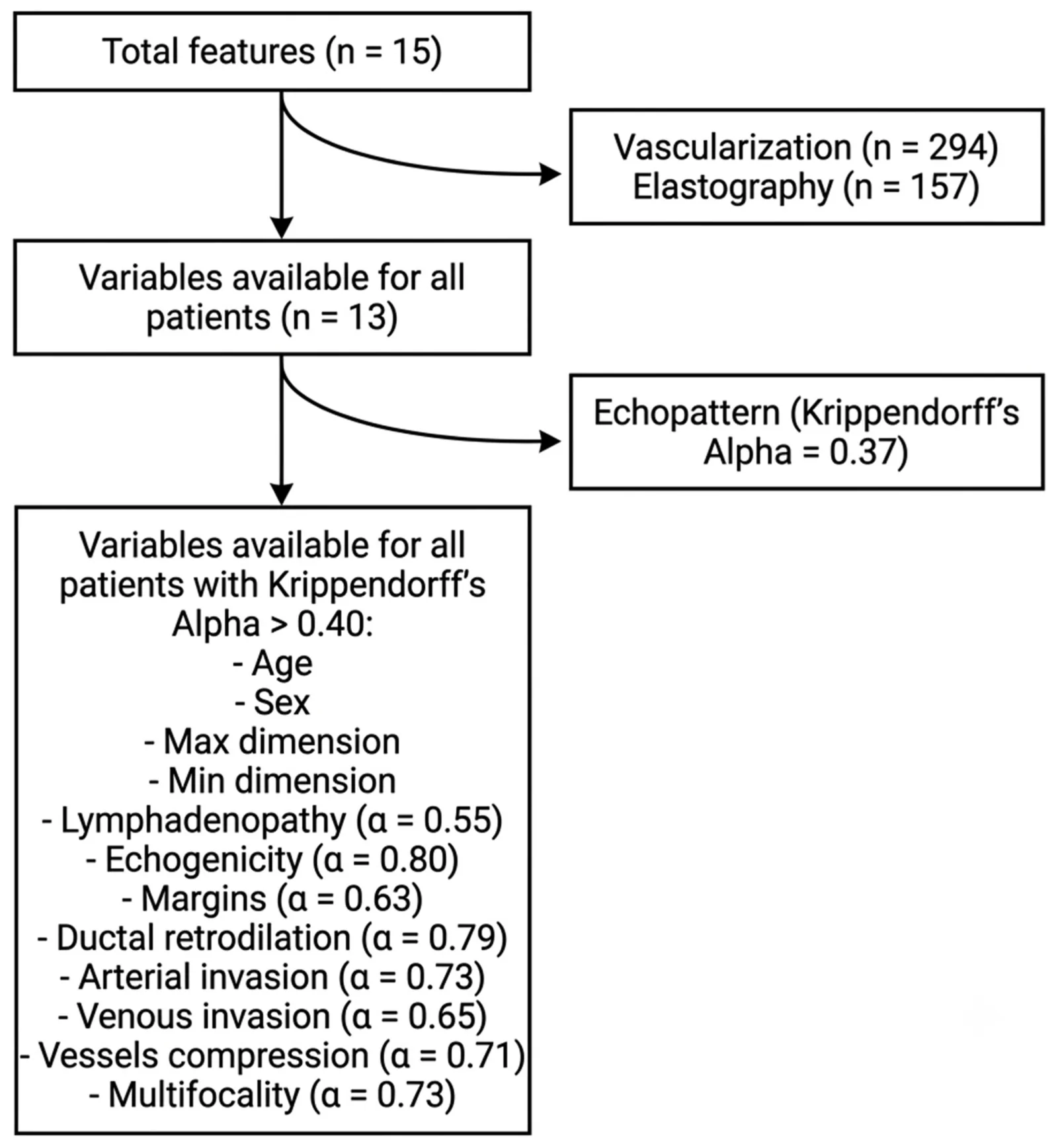
Inclusion–exclusion flow-chart: variables were selected based on sample size and inter-rater agreement.

**Figure 2:**
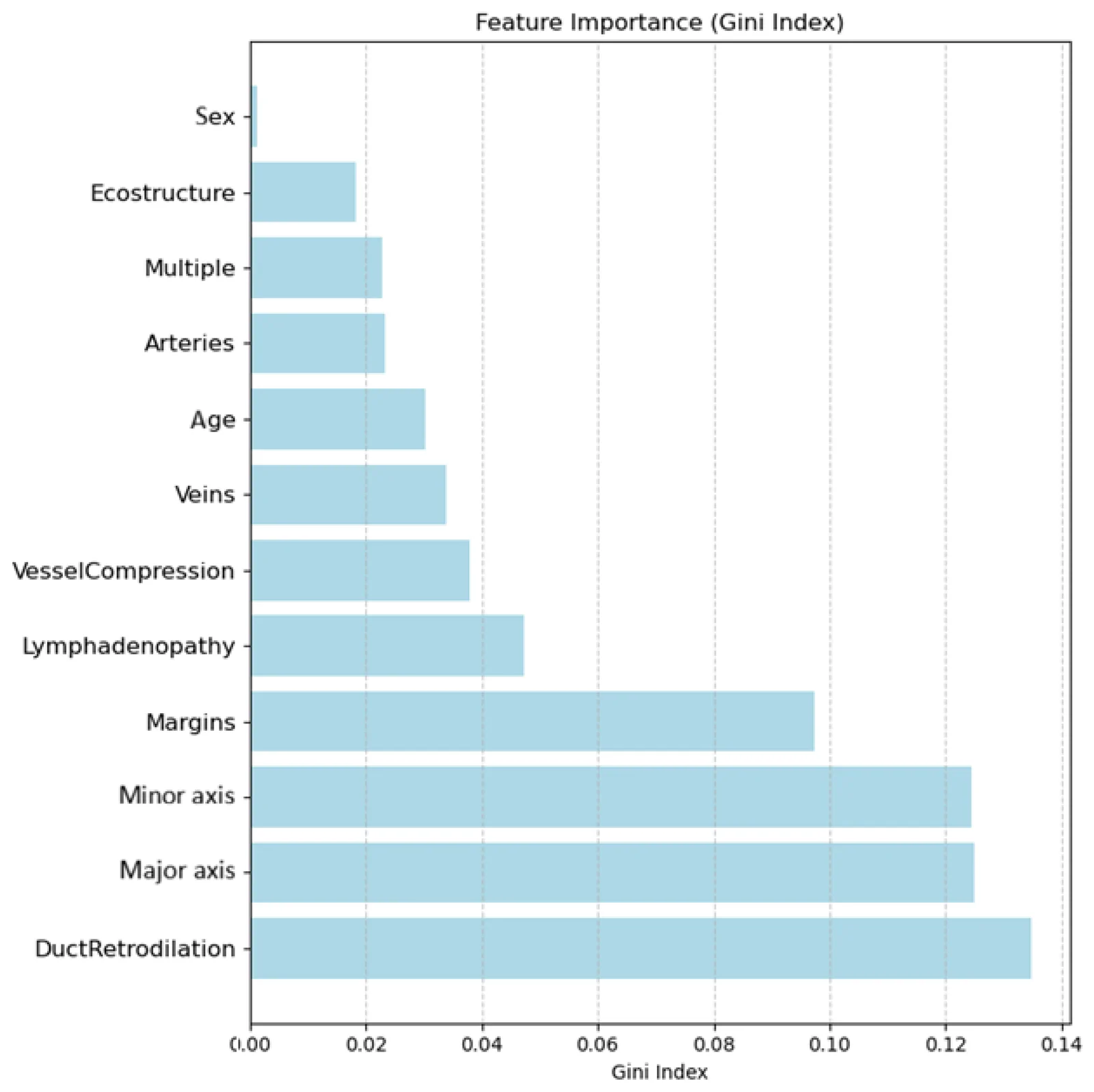
Feature importance computed through Gini Index gain.

The inclusion–exclusion workflow for the considered features is depicted in Figure 1. Figure 2 depicts the feature importance: for each feature, its association with the target variable (Diagnosis) is represented in terms of its contribution to reducing the Gini impurity of the target. To enable computation of weighted utility—and specifically to evaluate model performance in recognizing cases of different complexity and clinical relevance—one of the clinical authors also provided a complexity score for each instance of the test set.

### 2.3. Machine Learning Pipeline

The development pipeline was designed to support automated classification of PDAC versus non-PDAC cases using structured EUS-derived features. We adopted a modular pipeline architecture that encompassed hyper-parameter optimization (including data preprocessing), model construction and calibration, and performance evaluation. All steps were implemented in Python 3.11 using the scikit-learn, pandas, numpy, scipy, matplotlib and XGBoost libraries. The preprocessing stage included optional feature scaling (selectable among several methods including min-max scaling, max-abs scaling, standardization, normalization, Yeo–Johnson transformation, and robust scaling) and feature selection using Recursive Feature Elimination (RFE) with a logistic regression estimator 4. These steps were chained into Pipeline object from the scikit-learn package, and the specific methods used were selected through hyper-parameter optimization (see below). The following classifiers were evaluated: logistic regression (LR), k-nearest neighbors (KNN), decision tree (DT), Gaussian Naïve-Bayes (NB), support vector machine (SVM), random forest (RF), extreme gradient boosting (XGB) and a calibrated forest (CF). Each model was optimized and evaluated independently within a unified pipeline structure. Data from different hospitals were used for different purposes within the ML pipeline to ensure absence of bias and data leakage, as well as ensure generalizability of the obtained results. In particular, data from the Varese hospital was used as a hold-out external validation dataset, while data from the Policlinico and Brescia hospitals was used for training and model selection purposes.

### 2.4. Hyperparameter Optimization

Model hyperparameters were tuned using RandomizedSearchCV with 1000 iterations per model, applied on the training set (which consisted of data collected at the Policlinico and Brescia hospitals). A stratified leave one group out cross-validation strategy was applied within each optimization routine. The search space included both classifier-specific parameters (e.g., regularization strength C for LR, tree depth for DT and RF, learning rate and number of estimators for XGB) and pipeline parameters (e.g., number of features retained by RFE, scaling method and class imbalance management technique). The full list of hyper-parameters, along with the selected values, is reported in Table 1. The primary metric for model selection during hyperparameter tuning was balanced accuracy, chosen for its robustness to class imbalance. After hyper-parameter optimization, the optimal parameter set for each of the models was selected according to a variance-based penalization criterion [29], in order to limit the risk of overfitting on one specific cohort site. In particular, the balanced accuracy score reported by each parameter combination was penalized by the factor (1.96 σ)/√2, with this being the standard deviation across the two cohorts (Brescia and Policlinico) included in the training set. After selection, the optimal configuration of parameters was extracted and all classifiers were fitted on a training data created concatenating the data from Brescia and Policlinico. Final model performance was evaluated on the external hold-out test set (Varese).

### 2.5. Model Evaluation

Compared to more traditional studies, we evaluated our models using a broader and more comprehensive set of metrics, following the recommendations of the CHAMAI checklist [30]. In addition to evaluating discriminative performance, we also assessed calibration and utility. For discriminative performance, beyond the standard metrics, such as accuracy, sensitivity, specificity, positive predictive value (PPV), negative predictive value (NPV), area under the ROC curve (AUC), and F1 score, we included balanced accuracy and the F2 score, that is, respectively, the arithmetic mean of sensitivity and specificity and the harmonic mean of sensitivity and PPV, where the former is weighted twice as much as PPV, thus emphasizing recall over precision. Notably, we assessed whether the models provided sufficiently confident predictions, through the high-confidence (HC) counterparts of the above-mentioned metrics; this approach aimed to exclude cases where the model’s output was effectively a guess or only marginally informative. The high-confidence threshold, set at 70% (for the positive class, which corresponds to 30% for the negative class), was established in consultation with domain experts. Discriminative power was also graphically assessed by means of ROC and precision–recall curve analysis to analyze the variation in sensitivity and specificity (resp., PPV and sensitivity) at different prediction thresholds.

With respect to calibration, we evaluated the models’ ability to quantify uncertainty by measuring the agreement between the predicted probability scores and the observed (conditional) frequency of the target outcome. We adopted several standard metrics for calibration assessment: the Brier score [31], the Expected Calibration Error (ECE) [32,33], and the Estimated Calibration Index (ECI) [34]. Calibration was also evaluated visually using reliability curves. Utility assessment, in contrast, enabled us to evaluate the extent to which the developed models offer clinically meaningful support. This dimension was quantified using the Standardized Net Benefit (SNB) [35] and its recent extension, the weighted utility [36]. As also described previously, this latter metric required the construction of a test dataset in which each case was annotated according to its diagnostic complexity, thereby assigning greater weight to the model’s ability to correctly identify as positive (or avoid misclassifying as positive) the more diagnostically challenging cases, as compared to simpler and more common ones. Utility was also examined graphically through decision curves, which illustrate the variation in SNB across different prediction thresholds. All metrics evaluations were associated with 95% confidence intervals; for count-based metrics (accuracy, sensitivity, specificity, PPV, NPV, average precision, as well as the associated HC counterparts), confidence intervals were computed based on the binomial approximation method, whereas, for the remaining metrics, the formulas proposed in [37,38,39] were adopted.

## 3. Results

The multicenter design enabled external validation by training all classifiers on data from two sites (Milan and Brescia) and testing them on the remaining one (Varese), thereby improving their generalizability and evaluating the models under external validation conditions in the most conservative manner possible. Table 2 shows the diagnoses of solid lesions while Table 3 summarizes the performance metrics of all evaluated classifiers on the external test dataset, providing a comprehensive comparison across accuracy, discrimination, and utility measures. All values are reported with 95% confidence intervals.

Almost all classifiers achieved satisfactory performance, with only two (KNN and CF) reporting an accuracy lower than 0.85. Four classifiers, DT, RF, XGB and LR, reached an accuracy of 0.88. RF, XGB, LR, SVM and NB all reported sensitivity values higher than 0.90 (with the highest value, 0.98, reported by XGB), whereas DT achieved the highest specificity (0.85). The AUC score exceeded 0.80 for all classifiers and surpassed 0.90 for four of them (DT, RF, SVM, and NB). The selective prediction metrics were higher than the traditional ones for most models. For the three interpretable classifiers (DT, LR, and NB), performance improvements were consistent across all metrics. 

In particular, for DT and NB, these improvements were accompanied by relatively high coverage (greater than 0.80), indicating that focusing on highly confident predictions can enhance performance without reducing model’s usability.

Four models reported the best overall discriminative performance (DT, RF, LR, and NB), reaching scores ≥0.70 across all four confusion matrix metrics (i.e., sensitivity, specificity, PPV, and NPV), while achieving excellent values (>0.80) for sensitivity, AUC, F1, and average precision. Specifically, DT achieved high sensitivity (0.89), F1-score (0.92), and the highest balanced accuracy (0.87) and AUC (0.93), while maintaining transparency and interpretability, which are important considerations in the medical domain. RF, LR, and NB, by contrast, exhibited higher sensitivity values (>0.90), while yielding a lower specificity of, respectively, 0.70, 0.78 and 0.70. In particular, RF reported the highest F2 (0.93) and second highest sensitivity (0.94), while NB achieved the highest average precision (0.98). More generally, LR and NB reported similar performance to RF, while being as transparent and interpretable as DT.

### 3.1. Calibration and Utility

**Figure 3:**
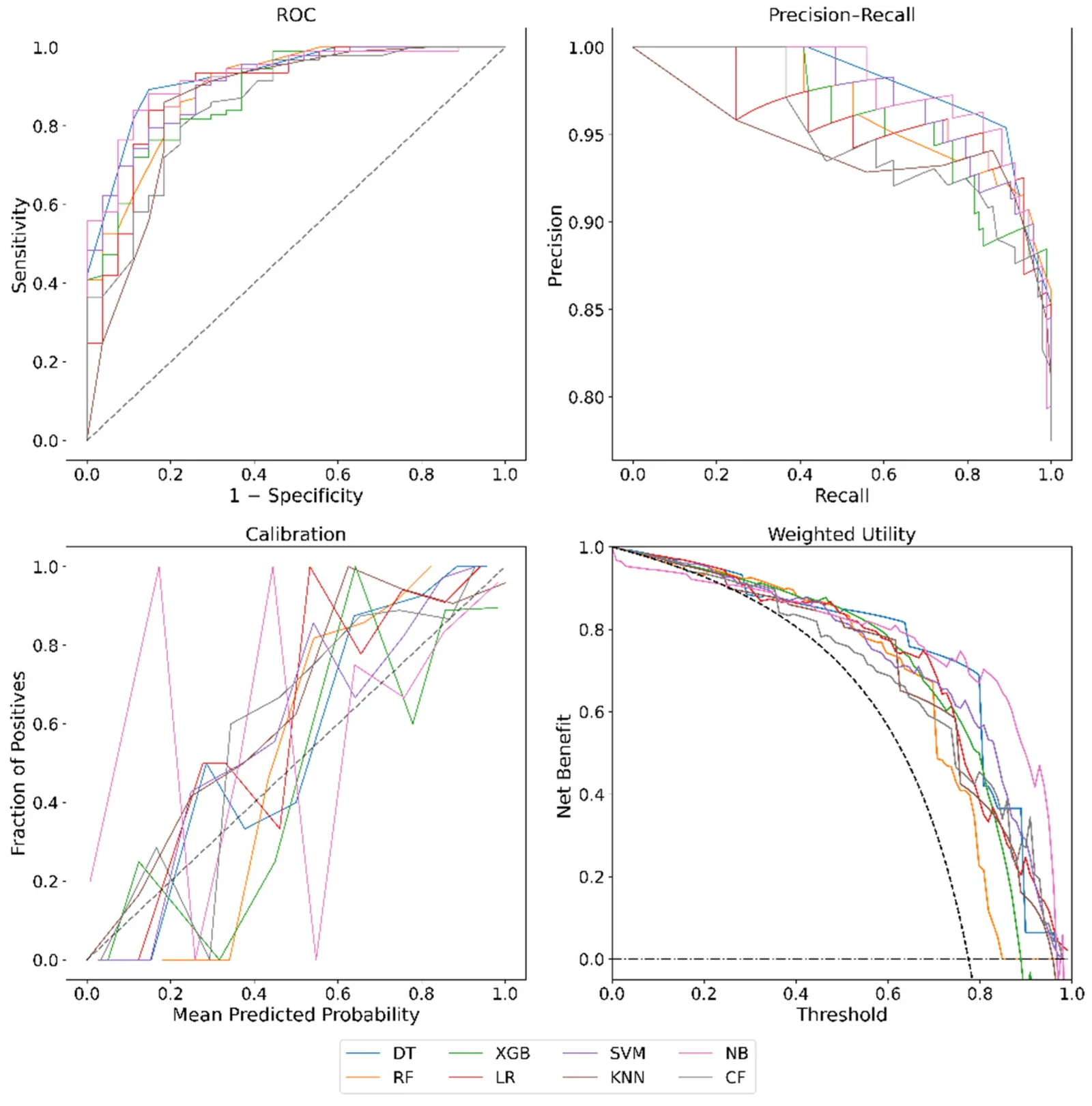
Comparative performance analysis of machine learning classifiers for PDAC diagnosis using structured EUS data. Top Left: receiver operating characteristic (ROC) curves for all classifiers. The decision tree (DT), support vector machine (SVM), and Naïve-Bayes (NB) models achieve the highest area under the curve (AUC), indicating strong discriminative power. Top Right: precision–recall (PR) curves highlighting the trade-off between positive predictive value (PPV) and sensitivity. RF, Naive Bayes (NB), and CF exhibit the highest average precision, maintaining strong performance under class imbalance. Bottom Left: calibration plots comparing predicted probabilities against observed outcomes. While none of the models are perfectly calibrated, DT, RF, and LR demonstrate relatively better alignment with the diagonal, supporting their clinical reliability. Bottom Right: (Standardized) net benefit curves for all classifiers.

In terms of the calibration metrics, all models reported satisfying performance, with a Global ECI (the higher the better) that goes from 0.79 to 0.94 and a Brier score (the lower the better) from 0.09 to 0.12. In particular, Naive Bayes reached the best ECI (0.94) and ECE (0.06), while DT obtained the best Brier score (0.09). Calibration curves (Figure 3) revealed that SVM, KNN, and DT showed relatively consistent alignment with the ideal calibration line. Miscalibration was more pronounced for XGB, RF and NB, which tended to strongly overestimate or underestimate probabilities in the mid-range. Similarly positive results were also reported in terms of utility, with all models having a standardized net benefit (SNB) higher than 0.75; RF, XGB and LR, in particular, reported the highest SNB value (0.85), though only CF reported a significantly lower value. RF also reported the highest weighted utility (WU) value (0.76). Interestingly, the values of WU were in all cases lower than SNB, indicating that the models may also face slight difficulties in correctly classifying clinically challenging instances.

### 3.2. ROC, PR and Decision Curves

To further assess model discrimination, calibration, and clinical utility, we conducted a comprehensive comparative evaluation using ROC curves, PR curves, calibration diagrams, and decision curve analysis (DCA). As shown in Figure 3, all models achieved high area under the ROC curve (AUC). Decision tree (DT, AUC = 0.93) demonstrated the highest discriminative performance, while RF, SVM and NB also achieved competitive AUC values (both ≥0.90), supporting their potential clinical applicability.

The PR analysis (Figure 3) highlighted differences in precision under class imbalance. NB achieved the highest average precision (A-PR = 0.98), indicating strong ability to maintain high positive predictive value across varying sensitivity levels. All models, and, in particular, DT, RF and LR, also performed well in this regard (A-PR ≥ 0.95).

Decision curve analysis (Figure 3) showed that all classifiers offered a net benefit greater than the “Treat All” and “Treat None” strategies across a wide range of decision thresholds (above 0.4). Although the NB model showed the lowest utility at low thresholds—where its curve fell below the “Treat All” baseline—its utility increased substantially for thresholds above 0.4.

**Figure 4:**
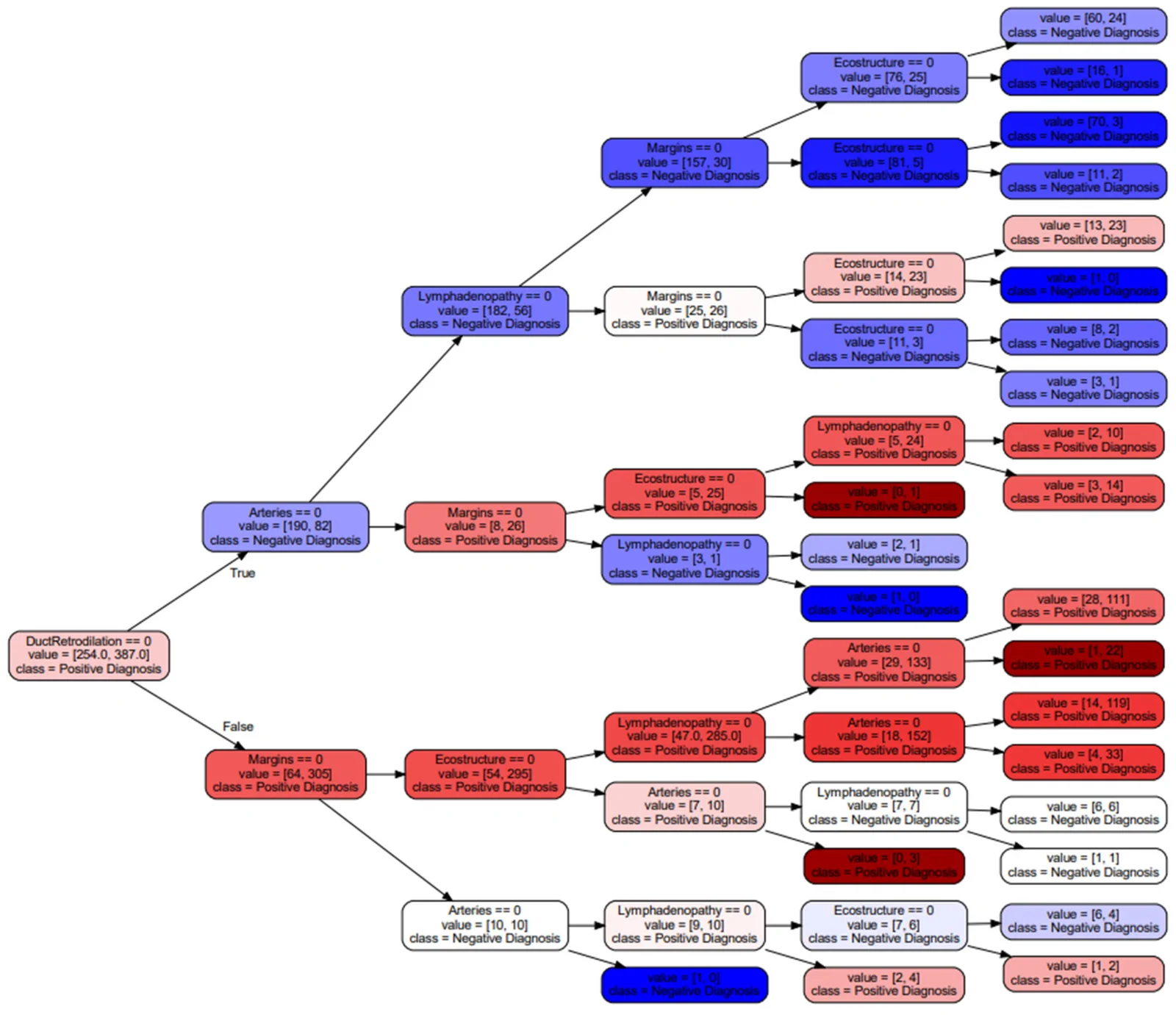
Decision tree plot. Each node depicts a split variable, with a corresponding threshold, the proportion of the two classes among the instances associated with the corresponding path connecting the root to the node, as well as the name of the prevalent class. Red color denotes a prevalence of PDAC cases, while blue color denotes a prevalence of the opposite class. White color denotes parity between the two classes.

To complement these findings, we report the constructed DT model, shown in Figure 4. The model identified duct retrodilation as the most important feature, followed by arterial invasion and margin regularity. Together, these features accounted for approximately 91% of the model’s total correct predictions.

## 4. Discussion

This study set out to determine whether machine learning (ML) models, and particularly interpretable ones, trained on structured features derived from routine endoscopic ultrasound (EUS) can accurately diagnose pancreatic ductal adenocarcinoma (PDAC). This objective addresses a critical gap in medical AI: the need for clinically deployable, transparent diagnostic tools, particularly in non-tertiary care settings. Unlike prior studies focused on raw imaging or radiomic features, which often entail substantial computational demands and limited interpretability, this investigation prioritized structured, human-interpretable variables already available in standard clinical workflows. A secondary aim was to assess whether the diagnostic performance of these interpretable models could approach or exceed the benchmarks reported in the literature for complex, less transparent models. The findings confirmed that interpretable classifiers such as decision trees (DT), logistic regression (LR), and Naive Bayes (NB) can achieve robust diagnostic performance. Among these, the DT model emerged as particularly promising, with an F1 score of 0.92 and sensitivity of 0.89, while maintaining high interpretability. These values match or exceed many performance metrics reported for more opaque models, demonstrating that performance and transparency need not be mutually exclusive. Importantly, the DT model also exhibited strong calibration and utility metrics, reinforcing its viability for clinical integration. Although ensemble methods like random forest (RF) and extreme gradient boosting (XGB) achieved the highest sensitivity, the trade-off in model complexity and interpretability, combined with the risk of overfitting (a limitation suggested by the low specificity of the XGB model) may limit their practical applicability. This reinforces the necessity of using balanced metrics—such as the F1 score and balanced accuracy—when evaluating models on imbalanced medical datasets. More broadly, in high-stakes clinical tasks, prioritizing sensitivity over specificity must be explicitly justified and aligned with clinical goals.

A substantial contribution of this work is its attention to calibration and selective prediction under uncertainty [40], assessed using advanced metrics such as Brier score, Expected Calibration Error (ECE), Estimated Calibration Index (ECI), and high-confidence (HC) metrics, which are fundamental to understanding how reliable the model’s predictions are. Calibration curves demonstrated that DT mostly maintained a monotonic relationship between predicted probabilities and actual outcomes—an essential condition for informed clinical decision-making. The HC metrics further illustrated that the most confident predictions made by these models were both reliable and accurate, while the inclusion of decision curve analysis (DCA) provided additional insights into the clinical utility of these models. DT, in particular, offered consistently positive net benefits across all decision thresholds, outperforming the default “Treat All” and “Treat None” strategies. This suggests that these models could effectively support clinical decision-making, particularly in ambiguous diagnostic scenarios, where the need for diagnostic support is greatest. The utility analysis thus bridges algorithmic performance with real-world relevance, an essential consideration in translational AI research.

Beyond model performance, the use of structured, clinician-curated features, unlike approaches requiring image processing or specialized hardware, allows for rapid implementation in clinical practice using existing data infrastructures, a significant advantage in resource-constrained settings.

Moreover, the availability of a dataset that clinicians can directly inspect enables additional analyses, such as descriptive and inferential statistics, useful for contextualizing their patient populations, which often differ across sites in geography and volume.

Several implications arise from these findings. First, they validate the use of interpretable ML as a viable alternative to complex models for oncological diagnostics, a critical attribute for serving as a decision-support tool for clinical specialists. Second, they highlight the importance of model calibration and uncertainty estimation for fostering clinical trust. Third, they suggest that decision-support tools based on structured data can be both powerful and practical, enabling broader integration into real-world workflows.

Nevertheless, an important limitation warrants attention: some endosonographic features were excluded because they were missing in a significant amount of reports, such as internal vascularization of the mass and elastographic evaluation, which could have improved the performance of the predictive models. This limitation is related to the retrospective design of the study, which therefore suffered from a lack of standardization in the writing of EUS reports in the description of a solid lesion of the pancreas. For analogous reasons, the calculation of inter-rater agreement should be interpreted with caution, particularly for certain features such as vessel invasion, as it was evaluated solely on still images, with no adequate video recordings available in the archives of the three centers.

Studies employing machine learning models for the diagnosis of pancreatic cancer remain limited, each characterized by its own distinct methodologies. The main ones are listed in Table 4.

It is worth noting that the performance of the models in the cited studies does not differ significantly from our results, despite their use of certainly more complex techniques and algorithms. In particular, the numbers from the study by Tian et al. and Kuwahara et al. [42,43], which shares our same objective (distinguishing the nature of a solid lesion), are analogous to our findings.

### Future Perspective

Our results underscore the importance of prospective validation and generalizability assessments in diverse clinical settings, which remain essential for regulatory approval and widespread adoption. In particular, future prospective studies should focus on addressing the limitations identified in our work, such as the quality and standardization of EUS reports. This would ensure the systematic collection of all critical ultrasonographic variables, including intralesional vascularity and lesion stiffness. 

Providing predictive models with more comprehensive datasets could significantly enhance their accuracy, thereby promoting their widespread adoption in routine clinical practice.

## 5. Conclusions

This study demonstrates that ML models trained on structured EUS features, rather than raw radiomics, offer a practical alternative for real-world clinical settings. By utilizing real-time clinical annotations, these interpretable classifiers achieve high diagnostic accuracy while ensuring transparency and compatibility with routine workflows. This approach facilitates a responsible transition from experimental AI to bedside tools, effectively augmenting clinical decision-making and setting a precedent for workflow-aligned AI integration in cancer diagnostics.

## Figures and Tables

**Table 1:** List of all the hyper-parameters evaluated for the developed models.

Model Hyperparameters	
Decision Tree	class weight: None, criterion: gini, max depth: 8, random state: 0, splitter: random, scaler: None, features to select: 5
Random Forest	class weight: None, criterion: entropy, max depth: 2, max feat: log2, n estimators: 720, random state: 0, scaler: None, feat to select: 9
Extreme Gradient Boosting	alpha: 0.455, eta: 0.123, gamma: 0.747, lambda: 2.370, max depth: 99, n estimators: 83, random state: 0, scale pos weight: 14.290, subsample: 0.801, feat to select: 10
Logistic Regression	C: 757.597, class weight: None, l1 ratio: 0.125, penalty: elasticnet, solver: saga, scaler: normalize, features to select: 8
Support Vector Machine	C: 789.539, class weight: None, gamma: scale, kernel: rbf, max iter:1000, probability:True, scaler: normalize, features to select: 11
k-Nearest Neighbors	algorithm: auto, n neighbors: 8, weights: uniform, scaler: min-max, features to select: 9
Naïve-Bayes	scaler: min-max, feat to select: 10
Calibrated Forest	criterion: gini, max depth: 84, max features: sqrt, n estimators: 981, random state: 0, method: isotonic, features to select: 11

**Table 2:** Count and percentages of diagnoses of solid lesions in the entire sample and in subgroups by center. PDAC: pancreatic ductal adenocarcinoma, NET: neuroendocrine tumors.

Diagnoses (*n*/%)	Total (*n* = 761)	Brescia (*n* = 475)	Milan (*n* = 166)	Varese (*n* = 120)
PDAC	480 (63.1%)	301 (63.4%)	86 (51.8%)	93 (77.5%)
NET	123 (16.2%)	70 (14.7%)	42 (25.3%)	11 (9.2%)
Metastases	40 (5.3%)	33 (6.9%)	2 (1.2%)	5 (4.2%)
Focal pancreatitits	27 (3.6%)	16 (3.4%)	6 (3.6%)	5 (4.2%)
Rare diagnosis	32 (4.2%)	20 (4.2%)	12 (7.2%)	0 (0.0%)
Non-diagnostic findings	59 (7.8%)	35 (7.4%)	18 (10.8%)	6 (5.0%)

**Table 3:** Evaluation metrics for each classifier. The table summarizes the main performance metrics for each classifier, along with corresponding 95% confidence intervals. Alternating row colors are used to enhance readability. DT: decision tree, RF: random forest, XGB: extreme gradient boosting, LR: logistic regression, SVM: support vector machine, KNN: k-nearest neighbors, CF: calibrated forest.

	DT	RF	XGB	LR	SVM	KNN	NB	CF
Accuracy	0.88 ± 0.06	0.88 ± 0.06	0.88 ± 0.06	0.88 ± 0.06	0.86 ± 0.06	0.85 ± 0.06	0.87 ± 0.06	0.82 ± 0.06
Sensitivity	0.89 ± 0.06	0.94 ± 0.05	0.98 ± 0.03	0.91 ± 0.06	0.90 ± 0.06	0.86 ± 0.07	0.91 ± 0.06	0.85 ± 0.07
Specificity	0.85 ± 0.13	0.70 ± 0.17	0.56 ± 0.19	0.78 ± 0.16	0.70 ± 0.17	0.81 ± 0.15	0.70 ± 0.17	0.70 ± 0.17
PPV	0.95 ± 0.04	0.92 ± 0.06	0.88 ± 0.06	0.93 ± 0.05	0.91 ± 0.06	0.94 ± 0.05	0.91 ± 0.06	0.91 ± 0.06
NPV	0.70 ± 0.16	0.76 ± 0.17	0.88 ± 0.15	0.72 ± 0.16	0.68 ± 0.17	0.63 ± 0.16	0.70 ± 0.17	0.58 ± 0.17
AUC	0.93 ± 0.01	0.90 ± 0.01	0.88 ± 0.01	0.89 ± 0.01	0.91 ± 0.01	0.86 ± 0.02	0.92 ± 0.01	0.86 ± 0.03
F1	0.92 ± 0.02	0.93 ± 0.02	0.93 ± 0.02	0.92 ± 0.02	0.91 ± 0.02	0.90 ± 0.02	0.91 ± 0.02	0.88 ± 0.03
F2	0.90 ± 0.02	0.93 ± 0.02	0.96 ± 0.02	0.92 ± 0.02	0.91 ± 0.02	0.88 ± 0.02	0.91 ± 0.02	0.86 ± 0.03
A-PR	0.97 ± 0.04	0.96 ± 0.04	0.96 ± 0.04	0.96 ± 0.04	0.97 ± 0.03	0.93 ± 0.05	0.98 ± 0.03	0.95 ± 0.05
Bal. Acc.	0.87 ± 0.06	0.82 ± 0.06	0.77 ± 0.07	0.85 ± 0.06	0.80 ± 0.06	0.84 ± 0.06	0.81 ± 0.06	0.78 ± 0.07
HC-Acc.	0.90 ± 0.05	0.94 ± 0.04	0.88 ± 0.06	0.93 ± 0.05	0.92 ± 0.05	0.89 ± 0.06	0.90 ± 0.05	0.92 ± 0.05
HC-Sens.	0.92 ± 0.06	1.00 ± 0.00	0.99 ± 0.02	0.96 ± 0.04	0.96 ± 0.04	0.92 ± 0.06	0.94 ± 0.05	0.97 ± 0.03
HC-Spec.	0.86 ± 0.13	0.67 ± 0.18	0.48 ± 0.19	0.78 ± 0.16	0.78 ± 0.16	0.77 ± 0.16	0.74 ± 0.17	0.71 ± 0.17
HC-PPV	0.96 ± 0.04	0.94 ± 0.05	0.88 ± 0.06	0.95 ± 0.05	0.95 ± 0.05	0.93 ± 0.05	0.93 ± 0.05	0.93 ± 0.05
HC-NPV	0.72 ± 0.15	1.00 ± 0.00	0.92 ± 0.13	0.82 ± 0.14	0.82 ± 0.14	0.74 ± 0.15	0.77 ± 0.16	0.86 ± 0.12
HC-Coverage	0.87 ± 0.06	0.72 ± 0.08	0.93 ± 0.04	0.79 ± 0.07	0.78 ± 0.07	0.81 ± 0.07	0.92 ± 0.05	0.71 ± 0.08
SNB	0.84 ± 0.01	0.85 ± 0.01	0.85 ± 0.01	0.85 ± 0.01	0.82 ± 0.01	0.83 ± 0.01	0.83 ± 0.01	0.76 ± 0.01
WU	0.74 ± 0.01	0.76 ± 0.01	0.75 ± 0.01	0.74 ± 0.01	0.70 ± 0.01	0.73 ± 0.01	0.72 ± 0.01	0.62 ± 0.01
Brier	0.09 ± 0.02	0.12 ± 0.01	0.11 ± 0.07	0.11 ± 0.03	0.11 ± 0.02	0.12 ± 0.04	0.10 ± 0.06	0.12 ± 0.04
ECE	0.09 ± 0.02	0.16 ± 0.01	0.07 ± 0.07	0.10 ± 0.03	0.09 ± 0.02	0.10 ± 0.04	0.06 ± 0.06	0.10 ± 0.04
ECI	0.86 ± 0.02	0.79 ± 0.01	0.90 ± 0.01	0.86 ± 0.03	0.87 ± 0.02	0.83 ± 0.04	0.94 ± 0.06	0.86 ± 0.04

**Table 4:** Summary of related studies in the literature, including their objectives, methodologies, and performance metrics. ML: machine learning, Sens: sensitivity, Spec: specificity, Acc: accuracy, AUC: area under the ROC curve, EUS: endoscopic ultrasound, SVM: support vector machine, ANN: artificial neural networks, CNN: convolutional neural networks, PDAC: pancreatic cancer adenocarcinoma, NET: neuroendocrine tumors.

Study	Objectives	ML Model	Performance Metrics			
			Sens	Spec	Acc	AUC
Zhang, 2010 [41]	Differentiating pancreatic cancer from normal tissue through EUS image analysis	SVM	0.94	0.99	0.98	-
Ozkan, 2016 [17]	Diagnose pancreatic cancer in a group of patients and healthy individuals using EUS image analysis, and test the models in subgroups based on age.	ANN	0.83	0.93	0.88	-
Tian, 2022 [42]	Diagnosing PDAC in a sample of solid pancreatic lesions of various types through EUS image analysis.	CNN	0.95	0.75	-	0.85
Kuwahara, 2023 [43]	Identify the nature of solid pancreatic lesions through the analysis of EUS images, testing models for each diagnosis, including PDAC.	CNN	0.94	0.82	0.91	0.90
Saraiva, 2024 [44]	Differentiating PDAC from NET by analyzing EUS images	CNN	0.99	0.84	0.94	0.94

## Data Availability

The data that support the findings of this study are available from Department of Pathophysiology and Transplantation, University of Milan but restrictions apply to the availability of these data, which were used under license for the current study, and so are not publicly available. Data are however available from the authors upon reasonable request and with permission of Department of Pathophysiology and Transplantation, University of Milan. The underlying code for this study is available in [MLEUS] and can be accessed via this link: https://github.com/MUDILab/MLEUS.git, accessed on 30 July 2026.

## Abbreviations

The following abbreviations are used in this manuscript:

AI	Artificial Intelligence
CF	Calibrated Forest
DT	Decision Tree
FNA/FNB	Fine Needle Aspiration/Fine Needle Biopsy
kNN	k-Nearest Neighbors
LR	Logistic Regression
ML	Machine Learning
NB	Naïve-Bayes
PDAC	Pancreatic Ductal Adenocarcinoma
RF	Random Forest
SVM	Support Vector Machine
XGB	XGBoost
